# Photopharmacology reveals high-specificity linkage of Ca^2+^ entry at TRPC6 nanodomains to NFAT activation in mast cells

**DOI:** 10.3389/fimmu.2025.1595036

**Published:** 2025-07-24

**Authors:** Denis Krivić, Olga Panasiuk, Johannes Pilic, Roland Malli, Bernadett Bacsa, Sanja Ćurčić, Klaus Groschner

**Affiliations:** ^1^ Division of Medical Physics and Biophysics, Gottfried Schatz Research Center, Medical University of Graz, Graz, Austria; ^2^ Division of Molecular Biology and Biochemistry, Gottfried Schatz Research Center, Medical University of Graz, Graz, Austria; ^3^ Core Facility Bioimaging, Center for Medical Research, Medical University of Graz, Graz, Austria; ^4^ BioTechMed-Graz, Graz, Austria

**Keywords:** TRPC6, photopharmacology, mast cells, basophils, Ca^2+^-transcription coupling, NFATc1

## Abstract

**Introduction:**

Photopharmacology has recently emerged as a strategy for high-precision modulation of immune functions. Here we explored efficiency and specificity of interventions based on light-induced TRPC6 activation in the RBL-2H3 mast cell model.

**Results:**

Expression of TRPC6 fusion constructs in RBL-2H3 allowed for generation of temporally well-defined, cytosolic Ca^2+^ transients in response to photoisomerization of the TRPC6 actuator OptoBI-1. These Ca^2+^ signals originated exclusively from Ca^2+^ entry across the plasma membrane. Transient TRPC6 activation in response to UV pulses of 1s duration (3 mW/cm^2^) just exceeded the detection threshold for monitoring of Ca^2+^ signals within the TRPC6-jRGECO1a nano/microdomain. Activation of TRPC6-jRGECO1a by single, 1s UV light pulses was sufficient to trigger maximal cytosolic to nuclear translocation of NFATc1 (NFAT2) equivalent to the level generated by ionomycin (1 µM)-induced Ca^2+^ entry. TRPC6 photopharmacology enabled control over NFATc1 nuclear translocation devoid of any detectable degranulation responses.

**Conclusion:**

We report here the exceptionally efficient and specific modulation of mast cell activity by TRPC6 photopharmacology.

## 1 Introduction

Mast cells, as well as basophils execute a range of diverse and incompletely understood physiological and pathophysiological functions, which are coordinated by highly specific Ca^2+^ signaling processes (1–10). As a key component of the innate and adaptive immunity, terminally differentiated mast cells and basophils are distributed throughout the body, residing in virtually all tissues with particular accumulation at sites that are exposed to or involved in interactions with the body’s external environment. Importantly, these cells serve not only as a first-line defense system against pathogens but represent enigmatic and complex players in mammalian reproduction, health maintenance as well as disease development and progression (4, 7, 8). As such, mast cells have recently emerged as an attractive, albeit ill-defined and challenging target for therapeutic interventions. Importantly, a complex, multifaceted role of mast cells has been proposed for tumor growth, neurodegeneration and cardiovascular as well as metabolic diseases, specifically the pathogenesis of diabetes (11–14). Mast cells communicate intensively with other immune as well as non-immune cells and determine the tissue microenvironment by the release of preformed, stored enzymes and mediators as well as by secretion of newly synthesized signaling molecules (4, 5). Their function as an immunological hub is for a large part coordinated by spatiotemporally well-defined cytoplasmic Ca^2+^ signaling patterns. A diverse set of Ca^2+^ channels, as well as transporters serve local signaling within cellular micro- or nanodomains to enable highly precise control over the immune cell´s Ca^2+^-secretion- and Ca^2+^-transcription coupling (3, 9, 15–18). Strategic positioning of Ca^2+^ channels into signaling nanodomains along with a channels´ ability to trigger temporally defined Ca^2+^ changes therein is considered as the basis of context-dependent tuning of immune cell functions. Spatiotemporally defined Ca^2+^ signals are selectively decoded by downstream Ca^2+^ sensors and transduced into a particular pattern of expression and secretion of cytokines, enzymes and mediators (10, 19, 20). A pivotal element involved in the transduction of mast cell Ca^2+^ signaling patterns into a cytokine expression profile are members of the NFAT transcription factor family. Transactivation of a specific set of NFAT proteins gives rise to a defined cytokine expression pattern thereby governing the immunological role of mast cells. We have recently observed that heterologously expressed TRPC6, a member of the canonical TRP channel family, can be operated by photopharmacology in a specific and temporally precise manner to control cell NFAT activity (21, 22). Based on the working hypothesis that “optical molding” of local Ca^2+^ signaling signatures in mast cells will enhance specificity and precision of therapeutic interventions, we set out to explore the suitability of TRPC6 photopharmacology for this concept. To do so, we utilized a recently established photochromic TRPC channel activator designated as OptoBI-1, which was found to be specific for TRPC3/6/7 (23). This compound has been characterized in depth in terms of its photopharmacological features (24) and demonstrated as a tool that enables control over both heterologously (22, 24), as well as endogenously expressed TRPC channels by light (23). We report robust TRPC6-mediated NFATc1 translocation in response to 1s activating light pulses associated with local Ca^2+^ transients barely exceeding the detection threshold. Our results demonstrate a high efficiency and specificity of pulsed photopharmacological modulation of TRPC6 in terms of transcriptional programming of mast cells.

## 2 Results

### 2.1 Low density plasma membrane expression of TRPC6 is sufficient to control CaN/NFATc1 signaling by brief photoactivation of the channel

**Figure 1:**
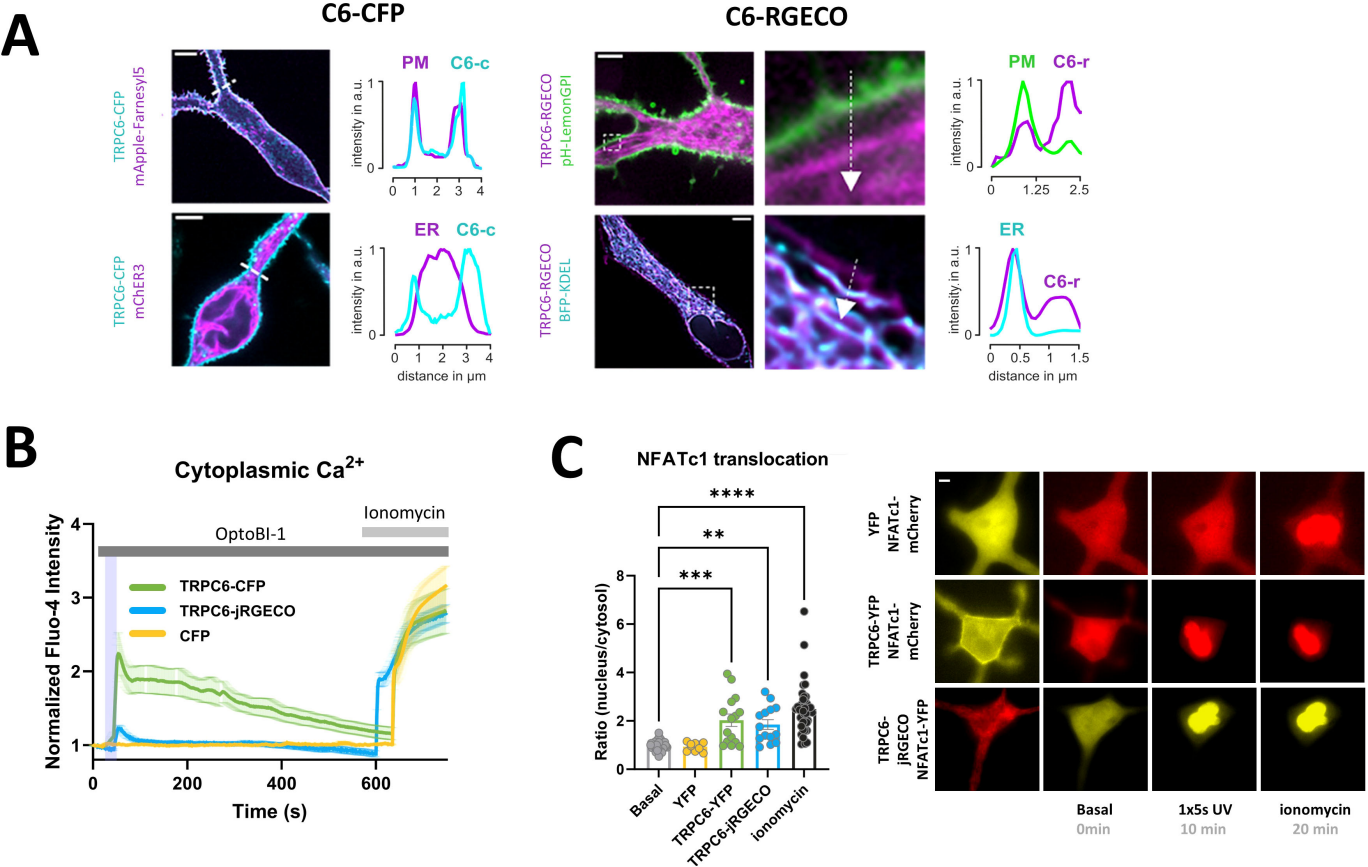
Low-level plasma membrane expression of TRPC6-jRGECO1a fusion channel is sufficient to enable robust photopharmacological activation of NFATc1 signaling in RBL-2H3 mast cells. **(A)** Analysis of the relative cellular localization of TRPC6 fusion proteins in the plasma membrane (PM) and the ER by confocal fluorescence imaging. Representative fluorescence micrographs with line scans of the intensity along indicated lines for TRPC6-CFP (C6-CFP) [left] and TRPC6-jRGECO1a (C6-RGECO) [right]. **(B)** Time courses of global Ca^2+^ signals measured as the increase in Fluo-4 AM fluorescence intensity in RBL-2H3 cells, which were genetically modified to overexpress CFP fluorescent protein (control; yellow; n=11) or TRPC6-CFP (green; n=16) and TRPC6-jRGECO1a (blue; n=18). Ca^2+^ entry via TRPC6 channel was initiated by 5s illuminations with UV light (365 nm, purple bar) 25s after addition of the photochromic TRPC3/6/7 activator OptoBI-1 (30 µM). 1 µM ionomycin was added to generate a reference signal, representing maximal cellular Ca^2+^ entry as indicated at 10min 20s. Mean values ± SEM are displayed. **(C)** Statistical representation of NFATc1 nuclear translocation triggered by the experimental condition shown in B at 10min after initiating Ca^2+^ entry by photoactivation. NFATc1 activation is quantified as the nucleus:cytosol fluorescence ratio of the co-expressed NFATc1-mCherry reporter fusion protein. Left: Bar graphs represent mean values ± SEM together with individual data points. Right: representative microfluorescence images. RBL-2H3 cells were genetically modified to overexpress YFP (yellow; n=10) or TRPC6-YFP (green; n=15) along with NFATc1-mCherry as a reporter for transcriptional activation. TRPC6-jRGECO1a (blue; n=14) was overexpressed together with NFATc1-YFP. The values designated as “Basal” represent the mean calculated from all three sets of experiments before photoactivation (0s), and “Ionomycin” designates the mean for the reference level calculated from all three conditions in cells exposed to OptoBI-1, UV light and ionomycin at the end of experiments (20min). Mean values ± SEM are shown. Statistical significance as assessed by one-way ANOVA with Dunnett’s *post hoc* test. Significance levels are indicated as: “ns” P > 0.05, **P ≤ 0.01, ***P ≤ 0.001, ****P ≤ 0.0001. All experiments were conducted in the presence of 2 mM extracellular Ca^2+^.

With this study, we set out to explore the technical suitability for high specificity control over mast cell function by TRPC photopharmacology. We hypothesized that light-mediated generation of distinct spatiotemporal Ca^2+^ signaling patterns will allow for enhanced specificity of interventions. As TRPC6 was recently identified as a suitable target channel for the manipulation of mast cell Ca^2+^ signaling and transcriptional activation by photopharmacology and chemo/optogenetics (22), we used TRPC6 overexpressing RBL-2H3 mast cells as a model system. TRPC6 activation in RBL-2H3 cells by brief (1s/5s) light pulses in the presence of the, photochromic actuator OptoBI-1 was confirmed in electrophysiological recordings (**Supplementary Figure 1**). In a first step, we characterized the cytosolic Ca^2+^ signals associated with brief photoactivation of two different TRPC6 fusion channels by use of OptoBI-1 as an actuator (23). Unexpectedly, we observed remarkable differences in the levels of plasma membrane expression for the TRPC-fusion constructs. One of the fusion constructs (TRPC6-jRGECO1a) is suitable for recording Ca^2+^ signals within the channel´s signaling domain, thereby enabling the monitoring of channel activity and local Ca^2+^ signaling in intact mast cells during photopharmacological interventions. **Figure 1A** illustrates the subcellular distribution jRGECO1a and plasma membrane targeting of TRPC-CFP and TRPC6-R-GECO. Confocal fluorescence imaging revealed an obvious difference in the propensity of these TRPC6 fusion channels to populate the plasma membrane (PM). TRPC6-jRGECO1a was mainly retained within the endoplasmic reticulum (ER) with only a minor fraction amounting typically to a fraction <10% that was targeted to the PM. By contrast, the TRPC6-CFP fusion showed prominent PM presentation, with only modest retention in the ER (**Figure 1A**). To compare the Ca^2+^ signaling capacity of these divergently targeted fusion constructs, we recorded global cytoplasmic Ca^2+^ signals (**Figure 1C**) from co-expressed cytosolic jRGECO1a. Consistent with the scarce PM targeting of TRPC6-jRGECO1a, cytosolic Ca^2+^ rises in response to its brief photoactivation were significant above controls (light-pulse or actuator only) but minute and only slightly above the threshold of detection, with peak levels amounting to about 20% of that recorded in TRPC6-CFP-expressing cells (**Figure 1B**). Interestingly, photoactivation of either TRPC6 fusion channel was found equally efficient in terms of transcriptional activation of NFATc1 representing a critical pathway of Ca^2+^-transcription coupling in immune cells (**Figure 1C**). The minute, global Ca^2+^ signal recorded upon activation of TRPC6-jRGECO1a channels by light was associated with robust activation of the CaN/NFATc1 pathway to a level comparable to its maximal activation by ionomycin (**Figure 1C**). **Supplementary Figure 2** depicts NFATc1 nuclear translocation triggered by photoactivation of TRPC6-CFP and TRPC6-jRGECO1a over the course of 60 min. This illustrates translocation kinetics including the maximum of activation. As the separate data sets (YFP, TRPC6-YFP and TRPC6-jRGECO1a) in **Figure 1C** exhibited similar basal- as well as ionomycin-induced reference values, the data were pooled, as represented in the two bars (grey and black). The additional rise in NFATc1 translocation generated by addition of ionomycin was significant for YFP and TRPC6-YFP (**** and * respectively), but not significantly different for TRPC6-jRGECO1a, as found by data analysis using individual data sets (not shown).

### 2.2 Photopharmacological control of mast cell NFATc1 activity via TRPC6 is exclusively based on Ca^2+^ entry through the TRPC channel

**Figure 2:**
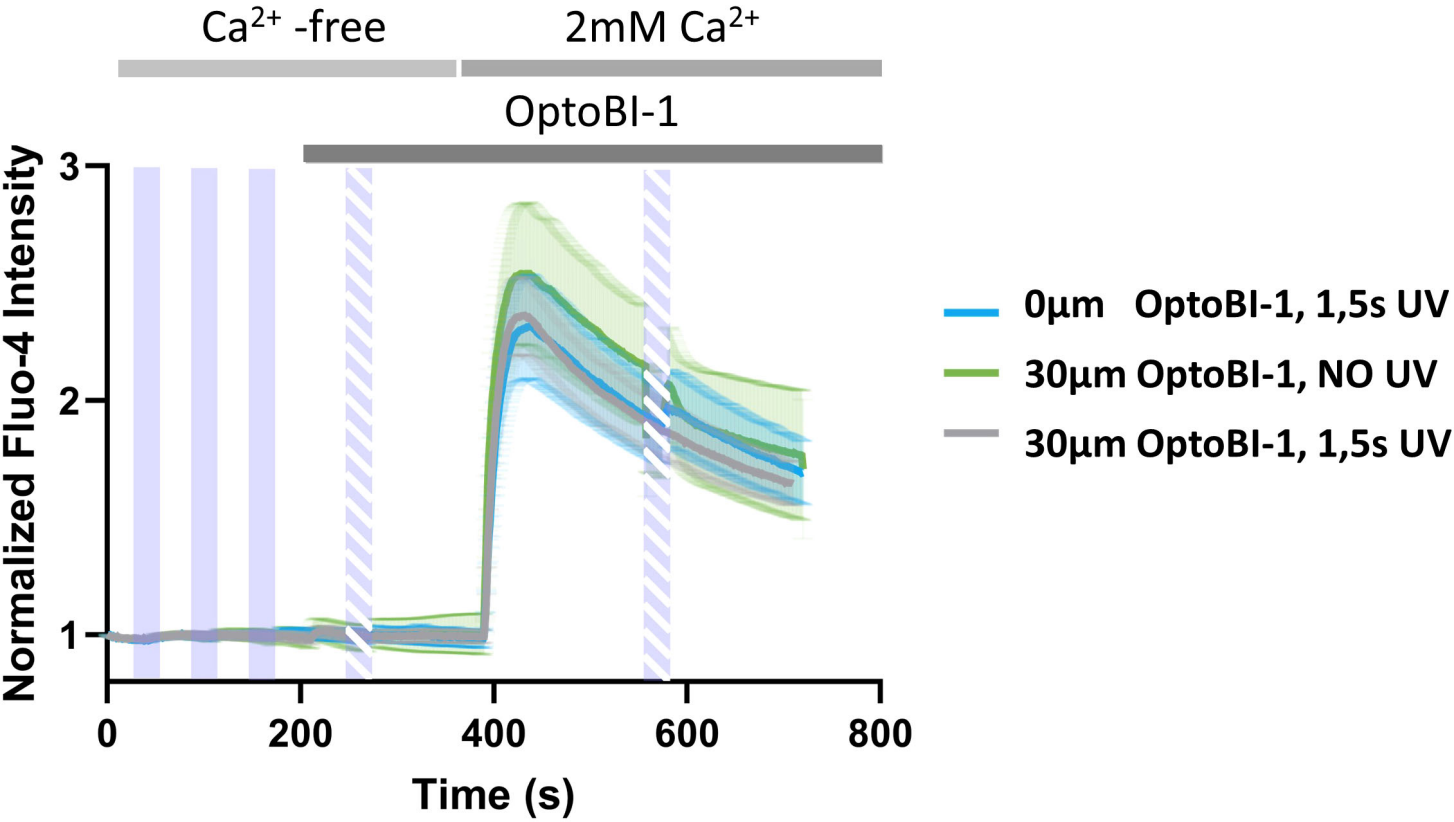
Photopharmacological activation of TRPC6-jRGECO1a fusion channels by OptoBI-1 neither mobilizes Ca^2+^ from the ER nor affects the endogenous SOCE pathway. Time courses of global Ca^2+^ signals measured as the increase in Fluo-4 AM fluorescence intensity in RBL-2H3 cells, which were genetically modified to overexpress TRPC6-jRGECO1a fluorescent protein. Cells were stimulated by 3x5s illumination with UV light (365 nm, purple bars) 45s after the start of the experiment, with 60s windows between the UV light pulses in nominally Ca^2+^-free conditions. Note that the exposure of RBL-2H3 cells to low extracellular Ca^2+^ initiates a partial, passive depletion of the ER, which results in significant Ca^2+^ entry upon extracellular Ca^2+^ re-addition via endogenous STIM/Orai1 complexes es evident in all experiments. Subsequently cells were exposed to the photochromic TRPC activator OptoBI-1 (30 µM) at 210s and TRPC6 was activated by 1x5s illumination with UV light as indicated (365 nm, dashed purple bar, (grey; n=13). Controls obtained with 1x5s illumination with UV light (365 nm, dashed purple bar) 30s in the absence of OptoBI-1 (blue; n=15), or by addition of OptoBI-1 (30 µM) at 210s without illumination with UV light (365 nm, dashed purple bar) (green; n=17) are shown. Ca^2+^ entry was initiated in all experimental conditions by addition of 2 mM Ca^2+^. Cells were stimulated one additional time with 1x5s UV light (365 nm, dashed purple bar) at 580s (grey and blue), in the presence of 2 mM extracellular Ca^2+^. Mean values ± SEM are displayed.

The predominant retention of TRPC6-jRGECO1a fusion channels within the ER compartment prompted us to test for a potential contribution of TRPC6-mediated disturbances of ER Ca^2+^ handling and an associated promotion of SOCE to the observed light-induced Ca^2+^ rises. To this end, we introduced our photoactivation protocol to cells exposed to a Ca^2+^ free extracellular solution and quantified store-operated Ca^2+^ entry (SOCE) via endogenous STIM/Orai complexes in the absence and presence of OptoBI-1-mediated activation of TRPC6 channels. To do so, we adopted a classical Ca^2+^-re-addition protocol. Initially, the cells were exposed to nominally Ca^2+^ - free buffer, which impedes Ca^2+^ entry and results in a partial, passive lowering of ER Ca^2+^ content that gives rise to a significant SOCE-mediated Ca^2+^ signal upon Ca^2+^ re-addition. As illustrated in **Figure 2**, the activation of TRPC6 channels in nominally Ca^2+^ - free extracellular solution failed to generate any detectable rises in Fluo-4 fluorescence, and SOCE, initiated by Ca^2+^ re-addition was not significantly altered by preceding TRPC6 photoactivation. This led us to conclude that the TRPC6-mediated Ca^2+^ signals and the associated translocation of NFATc1 as illustrated in **Figure 1**, were triggered by Ca^2+^ entry through plasma membrane-resident TRPC6 channels.

### 2.3 TRPC6 photoactivation triggers maximal NFATc1 nuclear translocation at threshold rises in the channel´s nanodomain Ca^2+^

**Figure 3:**
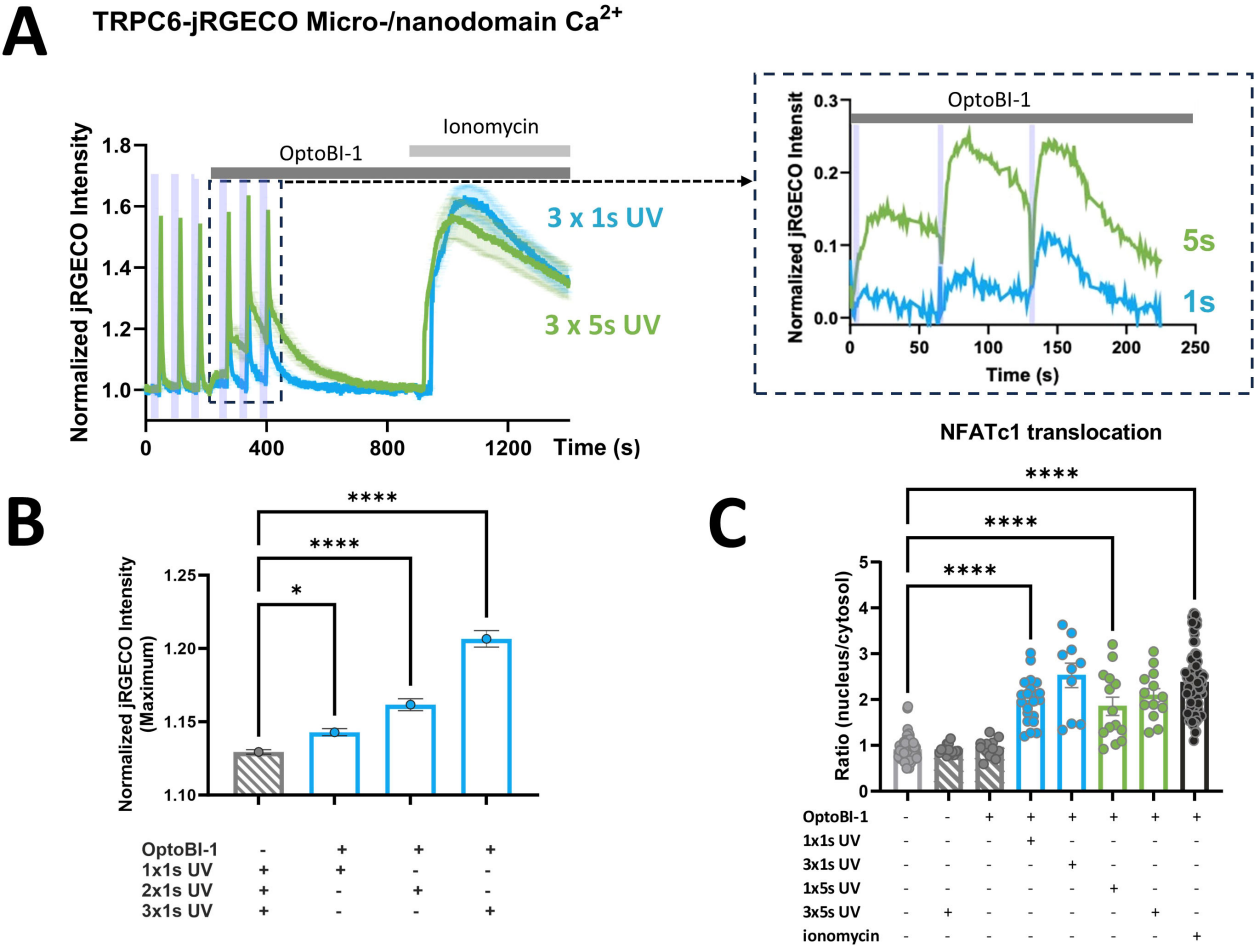
Ca^2+^ entry through TRPC6-jRGECO1a triggered by brief photoactivation that is threshold for detection by the channel-fused reporter, elicits maximal NFATc1 transcriptional activation. **(A)** Time courses of local Ca^2+^ signals measured as the increase in jRGECO1a fluorescence intensity in RBL-2H3 cells, which were genetically modified to overexpress TRPC6-jRGECO1a fluorescent protein along with NFATc1-YFP as a reporter for transcriptional activation. All cells were stimulated by 3x5s illumination with UV light (365 nm), with the first set of 3 illuminations (indicated by purple bars) 45s after the start of the experiment in the absence of OptoBI-1 in order to quantify the contaminating jRGECO1a photoswitching transients (control responses). Subsequently, the cells were exposed to the photochromic activator OptoBI-1 (30 µM) at 210s, and 3x1s (blue; n=16) or 3x5s (green; n=20) illumination with UV light (365 nm, second set of 3 illuminations) 30s after addition of OptoBI-1 (30 µM), with 60s windows between the UV light pulses. 1 µM ionomycin was added to generate a reference signal representing maximal cellular Ca^2+^ entry as indicated at 930s. Zoom-in (dashed rectangle) illustrates analysis of the OptoBI-1-induced Ca^2+^ signals by subtracting of the mean control responses representing jRGECO1a photoswitching artifacts from responses in the presence of OptoBI-1. **(B)** Statistical representation of peak jRGECO1a fluorescence intensity (maximum at 5s upon UV illumination) of the mean of the first three UV pulses, in the absence of OptoBI-1 (grey bar), and of peak jRGECO1a fluorescence intensity of subsequent pulses (blue bars) of the second set of three UV pulses, 1x1s, 2x1s and 3x1s UV, respectively. **(C)** Statistical representation of NFATc1 nuclear translocation triggered by the experimental condition shown in A at 10min after initiating Ca^2+^ entry by photoactivation. NFATc1 activation is quantified as the nucleus:cytosol fluorescence ratio of the co-expressed NFATc1-YFP reporter fusion protein. Bar graphs represent the mean values ± SEM together with individual data points. RBL-2H3 cells were genetically modified to overexpress TRPC6-jRGECO1a together with NFATc1-YFP. The values designated in the first (light grey) bar represent the mean level calculated from all six sets of experiments before photoactivation (0s), and the last (black) bar designates the mean for the reference level calculated from all six conditions in cells exposed to either OptoBI-1 or UV light or both, and ionomycin at the end of experiments (20min). Mean values ± SEM are shown. Statistical significance as assessed by one-way ANOVA with Dunnett’s *post hoc* test. Significance levels are indicated as: “ns” P > 0.05, *P ≤ 0.05, ****P ≤ 0.0001. All experiments were conducted in the presence of 2 mM extracellular Ca^2+^.

In the next step, we sought to delineate the rises in TRPC6-associated micro/nanodomain Ca^2+^ required for activation of the CaN/NFATc1 pathway. To obtain information on changes in Ca^2+^ levels within the TRPC6 harboring domain, we utilized jRGECO1a (RGECO) as a reporter fused to the TRPC6 channel. **Figure 3A** illustrates the time course of jRGECO1a signals recorded from RBL-2H3 cells during a sequence of brief UV light pulses (1 or 5s) in the absence and subsequent presence of the TRPC actuator OptoBI-1. Three consecutive light-triggered signals were recorded in the absence of the actuator to generate averages of the UV pulse-induced brief photoswitching transients typical for R-GECO (25). An averaged photostimulation artifact was used as a control for improved visualization and comparison of the Ca^2+^-dependent jRGECO1a signal (zoom-in in **Figure 3A**). Our analysis suggested that the generation of local Ca^2+^ rises at the TRPC6 channel surmounting the detection threshold of jRGECO1a, which requires photoactivation of the channel by UV pulses for a duration equal or longer than 1s. As illustrated in the insert of **Figure 3A**, 1s UV pulses generated minute jRGECO1a fluorescence transients above control. By contrast, 5s pulses were clearly associated with a significant Ca^2+^ entry-mediated jRGECO1a fluorescence. Considering the high sensitivity of jRGECO1a as a Ca^2+^ reporter (Kd < 200 nM), we concluded that the peak, local Ca^2+^ levels recorded in response to TRPC6 activation by a 1s UV pulse (at 30 µM OptoBI-1) would unlikely exceed 200 nM. To our surprise, these 1s UV pulses in turn not only triggered significant NFATc1 nuclear translocations but induced NFATc1 transcriptional activation (**Figure 3B**) comparable to the maximum reference level generated by ionomycin (1 µM). Of note, ionomycin controls were associated with profound and long-lasting elevation of micro/nanodomain Ca^2+^ levels (**Figure 3A**). Hence, our results show that a single, brief photoactivation of TRPC6 channels is capable of robust initiation of the CaN/NFAT signaling pathway in RBL-2H3 cells, despite barely detectable Ca^2+^ rises in the channel’s signaling nanodomain. Interestingly, when a train of short activating UV pulses was administered (3x1s), local Ca^2+^ rises were detected by the jRGECO1a reporter. An increase in local Ca^2+^ rises with pulse repetition was also observed with a pulse duration of 5s (**Figure 3A**). Importantly, pulse repetition-induced potentiation of the local Ca^2+^ responses was not associated with any changes in downstream NFATc1 activation (**Figure 3B**). In aggregate, these results suggest a remarkably tight coupling between TRPC6-mediated Ca^2+^ entry and NFATc1 nuclear translocation. It appears likely that this coupling involves in a first step an exceptionally efficient, spatio-temporal decoding of the Ca^2+^ entry signal by the downstream Ca^2+^/CaM/CaN signaling machinery within a specialized TRPC6 micro/nanodomain.

### 2.4 TRPC6 photoactivation in mast cells is not associated with immediate release of CD63-expressing granules

**Figure 4:**
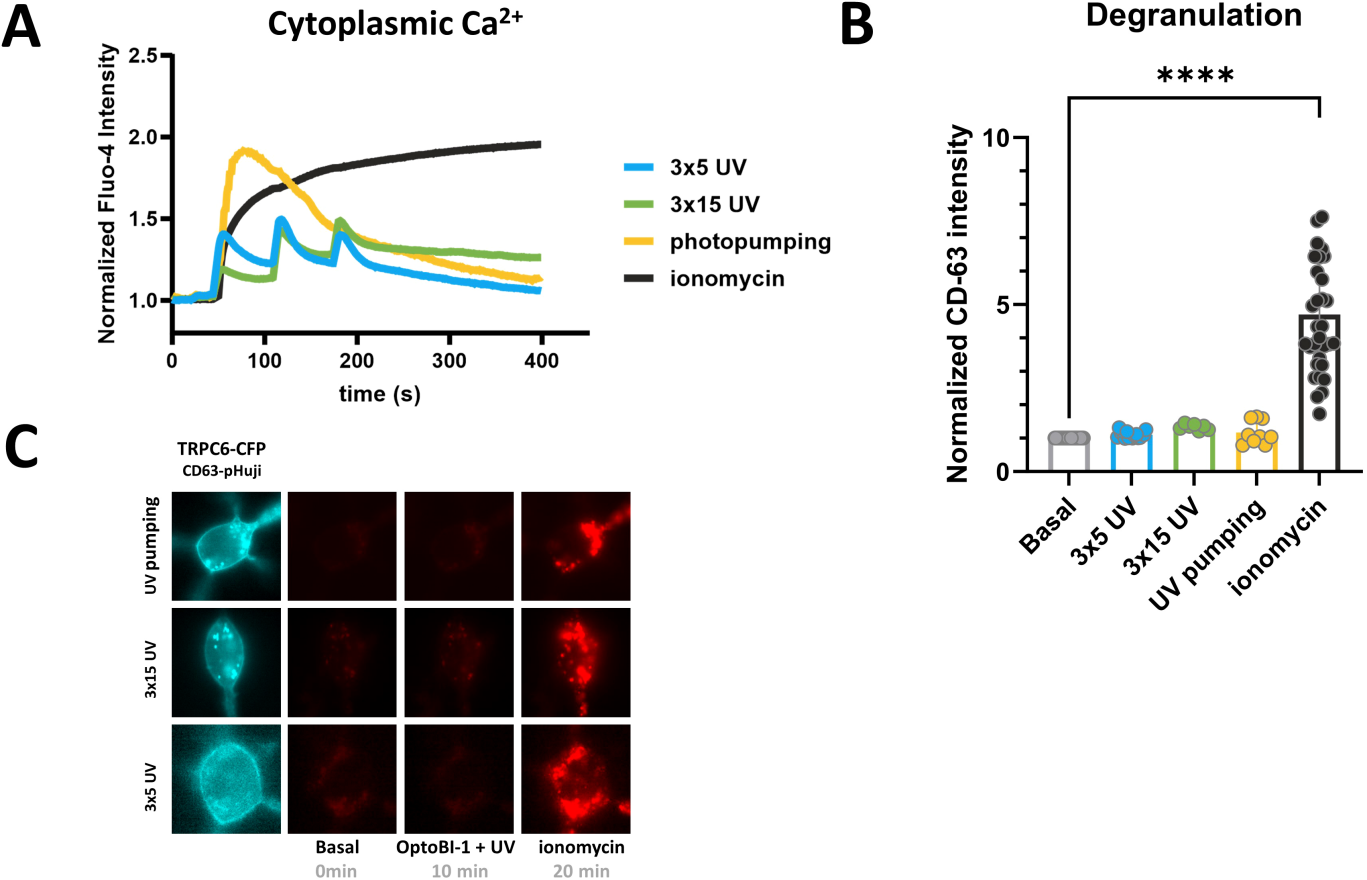
Light-controlled, prolonged TRPC6-activation is insufficient to trigger RBL-2H3 mast cell degranulation. **(A)** Time courses of global Ca^2+^ signals measured as the increase in Fluo-4 AM fluorescence intensity in RBL-2H3 cells, which were genetically modified to overexpress TRPC6-CFP fluorescent protein along with CD63-pHuji degranulation reporter. Ca^2+^ entry via TRPC6 channel was initiated by 3x5s (blue; n=14), 3x15s (green; n=7) and 30x3s (3s break between pulses) designated as photopumping (yellow; n=12) illuminations with UV light (365 nm, purple bar) 25s after addition of the photochromic TRPC activator OptoBI-1 (30 µM). 1 µM ionomycin was added to generate a reference signal representing maximal cellular Ca^2+^ entry as indicated at 10min 20s. Ionomycin response from the 3x5 experimental set is shown as a separate trace (black; n=14). Mean values ± SEM are displayed. **(B)** Statistical representation of CD63-pHuji intensity increase triggered by the experimental condition shown in A at 10min after initiating Ca^2+^ entry by photoactivation, 3x5s (blue; n=16), 3x15s (green; n=7) and 30x3s (yellow; n=9). RBL-2H3 mast cell degranulation is quantified as the normalized overall signal intensity increase of the co-expressed CD63-pHuji reporter fusion protein. Bar graphs represent the mean values ± SEM together with individual data points. RBL-2H3 cells were genetically modified to overexpress TRPC6-CFP along with CD63-pHuji as a reporter for mast cell degranulation. The values designated as “Basal” represent the mean level calculated from all three sets of experiments before photoactivation (0s), and “Ionomycin” designates the mean for the reference level calculated from all three conditions in cells exposed to OptoBI-1, UV light and ionomycin at the end of experiments (20min). Mean values ± SEM are shown. Statistical significance as assessed by one-way ANOVA with Dunnett’s *post hoc* test. Significance levels are indicated as: “ns” P > 0.05, ****P ≤ 0.0001. **(C)** Representative microfluorescence images. All experiments were conducted in the presence of 2 mM extracellular Ca^2+^.

As our findings suggested an exceptionally efficient translation of TRPC6 activation in terms of Ca^2+^ transcription coupling, we next asked whether TRPC6 Ca^2+^ entry is equally efficient in triggering mast cell degranulation as another essential cellular function of mast cells. Mast cell degranulation is a process that has been attributed to Ca^2+^ entry via TRPC channels (26), although evidence for such TRPC channel-mediated degranulation was typically obtained at scenarios of submaximal, long-lasting cytoplasmic Ca^2+^ rises (26). With the technology for light-mediated control of TRPC6 channels at hand, we were able to shape TRPC6-mediated cytoplasmic Ca^2+^ signals and analyze their impact on various amplitude and temporal features. As illustrated in **Figure 4A**, we generated repetitive (cyclic) Ca^2+^ transients with different amplitudes by varying the duration of channel activation, and also single maximal Ca^2+^ signals by high-frequency channel activation (“photopumping”). Although the amplitude of such maximal activation of TRPC6 channels by photopharmacology was equal to a reference maximum signal Ca^2+^ rise generated by the administration of ionomycin (1 µM), the entry through TRPC6 was insufficient to trigger detectable degranulation (**Figure 4B**). None of the temporal Ca^2+^ signaling patterns generated by TRPC6 photopharmacology was associated with degranulation of the RBL-2H3 mast cells, while the ionomycin-induced prolonged Ca^2+^ elevation triggered robust degranulation (**Figures 4B, C**). This clearly contrasts the profound TRPC6-mediated NFATc1 nuclear translocation, which was observed even close to threshold Ca^2+^ rises within the TRPC6 signaling nanodomain.

Our results demonstrate that TRPC photopharmacology is suitable for the generation of Ca^2+^ signaling patterns with defined amplitude and temporal features. We used this technology to reveal a remarkable downstream signaling specificity of TRPC6-mediated Ca^2+^ entry in RBL-2H3 mast cells.

## 3 Discussion

The ability of cellular Ca^2+^ signals to govern a wide array of cellular functions with high specificity relies on temporally well-defined Ca^2+^ signaling patterns generated within specialized signaling nano/microdomains. This led us to hypothesize that the exceptionally high spatiotemporal precision of manipulating cellular Ca^2+^ signals by photopharmacology might enable a new level of selectivity for the modulation of immune cell functions, which by far outperforms conventional pharmacological interventions. We focused on RBL-2H3 cells as a model of basophilic/mast cells, for which the feasibility of TRPC channel photopharmacology as an approach to modulate Ca^2+^ signals was recently demonstrated (21). As a pivotal component of the immune system serving a wide range of functions in human pathology, mast cells are considered as an attractive therapeutic target. Nonetheless, clinically useful modulation of mast cell function is challenging and inevitably requires the development of technologies for high-precision interventions. Our results corroborate the suitability of photopharmacology to precisely modulate mast cell transcriptional programing. In addition, we unravel a new mechanistic aspect of immune cell Ca^2+^ signaling and open the view on new concepts of high-precision immunomodulation.

In order to monitor the light-triggered Ca^2+^ signals in both the cytoplasm of RBL-2H3 as well as within TRPC6 signaling microdomains, we fused the Ca^2+^ reporter jRGECO1a to the N-terminal tail of TRPC6. Unexpectedly, we observed a dramatically reduced plasma membrane presentation of the fusion construct compared to conventional N-terminal GFP fusions of the TRPC channel. Correspondingly, global cytoplasmic Ca^2+^ signals evoked by photopharmacologic activation were minute in comparison to those triggered in cells expressing the well-plasma membrane targeted TRPC6-CFP fusion channel (**Figure 1**). To our surprise, TRPC6-jRGECO1a - expressing cells displayed a remarkably robust, close to maximum, translocation of NFATc1 in response to photactivation. The UV light-triggered TRPC6 activation was based on the photochromic activator OptoBI-1, which was demonstrated as specific for the primarily lipid-gated TRPC subfamily (TRPC3/6/7) (23). RBL-2H3 typically expresses low levels of TRPC6 transcripts (21), while significant expression of TRPC3 and TRPC7 mRNA has been reported (26). Control experiments with CFP (sham)-transfected RBL-2H3 indicated a lack of endogenous OptoBI-1-sensitive TRPC6 channels. This led us to conclude that RBL-2H3 cells do not express a significant functional background of endogenous TRPC3/6/7 containing Ca^2+^-permeable channels. Importantly, the heterologous expression of a TRPC6 fusion protein, which is only scarcely presented in the plasma membrane, enabled Ca^2+^ signaling that was highly effective in terms of NFAT transcriptional activation. As the TRPC6-jRGECO1a fusion channel was significantly retained in the ER, and a function of its relative TRPC3 in the ER membrane has previously been suggested (27), we were prompted to test if the observed TRPC6-dependent NFAT activation might involve altered ER function or promotion of SOCE. Experiments performed in Ca^2+^-free extracellular solutions and the evaluation of SOCE in classical Ca^2+^ re-addition protocols indicated that activation of overexpressed TRPC6-jRGECO1a neither triggered depletion of the ER Ca^2+^ store nor promoted the RBL-2H3 SOCE pathway. From these findings, we concluded that the observed OptoBI-1/light-mediated transcriptional activation of NFATc1 was exclusively driven by Ca^2+^ entry through plasma membrane resident TRPC6 channels.

### 3.1 High-efficiency translation of TRPC6-mediated Ca^2+^ entry into NFATc1 signaling

By utilizing the power of photopharmacology in terms of spatiotemporal precision of target activation, we set out to further characterize the efficiency of TRPC6-mediated Ca^2+^-transcription coupling. To this end, we recorded local Ca^2+^ rises at the TRPC6 signaling domain along with NFATc1 translocation at low, close to threshold levels of light-triggered TRPC6 activation. A well-defined, graded TRPC channel activation was feasible by varying the duration of the activating UV light pulses. Thereby, we determined a threshold of about 1s illumination for the detection of significant Ca^2+^ rises by the jRGECO1a reporter fused to the TRPC6 channels. It is important to note that activation of a small but significant TRPC6 conductance was clearly detectable by patch-clamp recordings in response to brief (1s) pulses of UV light for TRPC6-YFP fusion channels (**Supplementary Figure 1**). In these conventional whole-cell recordings with cytoplasmic Ca^2+^ - buffered by EGTA, the UV light-induced TRPC6 currents were fairly stable over time, with only little enhancement during repetitive activation. Our recording of the local Ca^2+^ rises, generated in the channel vicinity of intact cells, revealed that the UV light-induced, local Ca^2+^ signals were transient, lasting for about 1min. Our electrophysiological recordings were consistent with a threshold for light/OptoBI-1-induced TRPC6 activation at about 1s UV pulse duration. Interestingly, repetitive channel activation by UV illumination resulted in gradually increasing local Ca^2+^ transients, while TRPC6 membrane conductance remained surprisingly stable during the repetitive photoactivation protocol (**Supplementary Figure 1**). This indicates the existence of a process that potentiates the local Ca^2+^ rises, independently of changes in channel activity during repetitive TRPC6 activation. It is tempting to speculate about a local reorganization of membrane structures and strategic positioning of Ca^2+^ handling elements during prolonged TRPC6 activation. The mechanistic basis of this potentiation remains to be clarified. Nonetheless, this phenomenon also enabled the light-controlled generation of defined, longer-lasting cellular Ca^2+^ responses by appropriate temporal illumination protocols (photopumping). Interestingly, a single, brief (1s) photoactivating stimulus, at the threshold for detecting TRPC6 activity in the membrane of RBL-2H3 cells, was sufficient for NFATc1 transactivation corresponding to the maximum reference response achieved by ionomycin-induced Ca^2+^ entry. Considering the rather high sensitivity with a reported Kd of around 200 nM of the Ca^2+^ reporter jRGECO1a (28, 29), our results suggest that even modest, local Ca^2+^ rises in the range of <200 nM, confined to the TRPC6 signaling nano/microdomain, as recorded with 1s UV pulses, are sufficient to trigger full activation of the calcineurin-NFAT pathway in RBL-2H3 cells. Hence, our findings suggest TRPC6 as part of a signaling domain that allows for exceptionally efficient translation of Ca^2+^ entry into NFAT activation. Our present study focused specifically on the NFATc1 (NFAT2) isoform. This transcription factor is prominently expressed in basophils (30) and serves important functions throughout the immune system, including also mast cells, where it was found to control cytokine production in cooperation with its NFATc2 (NFAT1) (31). It appears reasonable to speculate that TRPC6 may similarly control other NFAT species, specifically its closer relative NFATc2. Moreover, Ca^2+^-dependent transcriptional regulation in immune cells is typically based on integration of multiple pathways including also NFkB signaling. NFkB is activated in a complex manner by multiple Ca^2+^-dependent processes, and reportedly serves as a co-regulator of immunological responses along with NFAT (32). Hence, future studies will be required to analyze in greater detail how other processes of Ca^2+^ transcription coupling are linked to TRPC6 activity.

### 3.2 High-specificity coupling between TRPC6-mediated Ca^2+^ entry and NFATc1 activation

The specificity of photopharmacological control over Ca^2+^-transcription coupling was demonstrated by the complete lack of mast cell degranulation in response to TRPC6-mediated Ca^2+^ entry. Of note, TRPC3 as well as TRPC5 are reportedly able to generate pro-inflammatory Ca^2+^ signals in mast cells (15, 26), while a contribution of TRPC6 in mast cell activation has been questioned (33). The authors considered insufficient plasma membrane presentation as a potential one reason for the observed failure of endogenous TRPC6 to generate productive Ca^2+^ signals in human mast cells (33). Here we show that the specific coupling of TRPC6 Ca^2+^ signaling to transcriptional activation of RBL-2H3 cells does not require prominent expression and membrane presentation of TRPC channels. To our surprise, specificity for CaN/NFAT signaling vs. degranulation was not based on the temporal precision of TRPC6 activation and the generation of a distinct temporal Ca^2+^ signaling pattern. Even maximal, long-lasting activation of TRPC6 Ca^2+^ entry by a continuous, high-frequency photoactivation protocol (photopumping) failed to initiate significant degranulation. Of note, applied high-frequency photoactivation protocol did not generate an entirely stable, long lasting TRPC6-mediated Ca^2+^ rises. We can therefore, not conclusively distinguish if the observed signaling specificity with lack of degranulation was based on the still transient Ca^2+^ dynamics, or on the targeting of TRPC6 channels into a specialized signaling domain that is not linked to exocytotic machinery. Nonetheless, we conclude that heterologously expressed TRPC6 channels, even when residing in the plasma membrane at a strikingly low density, are endowed for highly efficient control of NFAT transcriptional activation, while these channels are largely uncoupled from the Ca^2+^-dependent exocytotic machinery of degranulation. We propose that this might also apply to endogenous TRPC6, giving rise to specific TRPC6-mediated Ca^2+^ transcription coupling at essentially low density of the TRPC channel in the plasma membrane. Hence, it appears important to consider functional relevance of even minor alterations in TRPC6 expression, which may occur in certain immunological or pathophysiological settings.

Such high-efficiency and high-specificity signal transduction within plasma membrane micro/nanodomain has been suggested to involve spatial precision of signal transduction, based on strategic positioning of the communicating signaling elements, as well as confinement of the signaling space by specialized membrane structures. This scenario enables fast transfer of Ca^2+^ from its source to the Ca^2+^ decoding target as proposed for Ca^2+^ signaling in ER-PM nanojunctions (34). It remains to be clarified if TRPC6 channels are targeted into specialized membrane architectures such as PM-organelle junctions.

Currently, we can only speculate about the mechanistic basis of the observed high efficiency and specificity of NFAT activation triggered by TRPC6 photopharmacology in RBL-2H3 cells. Nonetheless, we propose that mast cells and basophilic cells feature specialized membrane structures capable of integrating TRPC6 as a Ca^2+^ entry channel for exceptionally efficient Ca^2+^ transcription coupling. We conclude that even barely detectable changes in the TRPC expression or membrane recruitment, may dramatically alter the Ca^2+^ handling and function of these multifunctional immune cells. Therefore, current concepts envisioning TRPC channels as therapeutic targets may deserve a careful reconsideration and extension (35–38). Our study further substantiates the value of photopharmacology and pharmacogenetic technologies as a novel approach for high-precision modulation of immune cell Ca^2+^ - transcription coupling and function. Experimentally, this strategy may enable deeper insights into the complex functions of mast cells and basophils, and pave the way towards therapeutically valuable interventions.

## 4 Materials and methods

All reagents used were of molecular biology grade, purchased from Merck KGaA (Darmstadt, Germany) unless specified otherwise. pGP-CMV-NES-jRGECO1a (#61563), pCMV-Sport6-CD63-pHuji (#130902), BFP-KDEL (#49150), mApple-Farnesyl-5 (#54899) and mCherry-ER-3 (#55041) plasmid DNA constructs were obtained from Addgene.

All plasmid DNA constructs (available upon request) used for protein overexpression in experiments are stored locally and designated as follows (excluding the above-mentioned ones): pe-CFP-C1, pe-YFP-C1, hTRPC6 in peCFP-C1, hTRPC6 in peYFP-C1, hTRPC6-jRGECO1a in pEF1a, mCherry-Linker-NFAT-C1 in PAD4, YFP-NFAT-C1 and pH-LemonGPI.

### 4.1 Cell culture and transfection

RBL-2H3 cells were cultured in 10 cm dishes in Dulbecco’s Modified Eagle Medium (DMEM low glucose, D5546, Invitrogen) with 10% supplement of fetal bovine serum (FBS), L-glutamine (2 mmol/L), streptomycin (100 μg/mL), and penicillin (100 U/mL) at 37°C and HEPES (10 mmol/L), and 5% CO_2_ level. Cells were authenticated by short tandem repeat (STR) analysis and regular tests were performed to confirm the lack of contamination with mycoplasma. For gene introduction for electrophysiology and microscopy experiments, the media was aspirated and RBL-2H3 cells were rinsed with PBS. Cells were incubated with accutase (3 mL) for 10min at 37°C. The cells were centrifuged at 600 G for 4min, the supernatant was discarded and the cells were re-suspended in Opti-MEM reduced serum medium. Afterwards, the cells were counted using the (Luna-II; Logos Biosystems; BioCat) automated cell counter, and the amount of Opti-MEM was adjusted to the final cell suspension with concentration of 1x10^5^ - 1x10^7^ cells/mL. The cell suspension was split into 1.5 mL tubes with 400 μL per tube. Plasmid DNA was added to each tube as required (5 μg per construct). Thereafter, the cells were transferred into 4-mm electroporation cuvettes (Bio-Rad, US). The electroporation was performed by applying 200 V and 950 μF in a Gene Pulser II (Bio-Rad, UK). Upon electroporation, 400 uL of the full cell culture medium was added to cells and the cells were transferred from the cuvettes into 3.5 cm dishes containing cell culture medium and coverslips. Experiments were performed 18–24h after electroporation.

### 4.2 Ca^2+^ imaging

Briefly, RBL-2H3 cells transiently overexpressing target proteins seeded onto coverslips were kept in Tyrode’s buffer containing (in mmol/L) 140 NaCl, 5 KCl, 1 MgCl_2_, 10 HEPES, 10 glucose and 1 CaCl_2_, pH = 7.4 for 10-30min before the start of each experiment. If Fluo-4 AM was used as a Ca^2+^ reporter, the cells were incubated in 2 μM of the dye at room temperature for 30min, washed three times with the Tyrode’s buffer solution and left for an additional 10min period in the solution before measuring. Subsequently, a coverslip was transferred to a measuring chamber containing the same type of solution on an inverted microscope (Olympus IX71, Vienna, Austria) with 60 × 1.3 N.A. oil-immersion objective. Changes in intracellular Ca^2+^ ([Ca^2+^]i) were monitored by excitation of red-shifted genetically-encoded Ca^2+^ sensor (jRGECO1a) using 577/25 nm filters via TILL Oligochrome light source (TILL Photonics FEI Company, Graefelfing Germany). Fluorescent images were captured every half-second at 632 nm (using 632/60 nm emission filter, Chroma Technology, VT, USA) with an ORCA-03 G digital CCD camera (Hamamatsu, Herrsching am Ammersee, Germany) using Live Acquisition 2.6 software (TILL Photonics FEI Company, Graefelfing Germany). The values acquired (Fluo-4 AM or jRGECO1a absolute intensity), and if necessary corrected, were then plotted using the GraphPad Prism software V10.0 (Systat Software Inc.).

The activation of OptoBI-1 compound was achieved with 365 nm light exposure for 1, 3, 5 or 15s period. The illumination was repeated multiple times depending on the experimental setup. The interval between each illumination onset of a train of light pulses was 60s, unless otherwise specified. All experiments were performed at room temperature.

### 4.3 Quantification of NFAT nuclear translocation and degranulation

Translocation of NFAT in NFATc1-mCherry or NFATc1-YFP used as a control or in tandem with TRPC6-CFP and TRPC6-jRGECO1a overexpressed in RBL-2H3 cells was observed using an inverted microscope (Olympus IX71, Germany) equipped with a 60 × 1.3 NA oil immersion objective. During the recordings using Live Acquisition v2.6 software (TILL Photonics FEI Company, Gräfelfing Germany), the excitation of mCherry was achieved using 577/25 nm filter and fluorescent images were captured every 15s at 632 nm (using 632/60 nm emission filter Chroma Technology, VT, USA) with an ORCA-05G digital CCD camera (Hamamatsu, Herrsching am Ammersee, Germany). ImageJ 1.51n software was used to measure the fluorescence intensity in the nucleus and cytoplasm before and after stimulation with given concentration of OptoBI-1. These values (nucleus/cytosol) were then plotted using the GraphPad Prism software V10.0 (Systat Software Inc.). The translocation of NFATc1 in RBL-2H3 cells was observed for 20min, unless otherwise specified.

### 4.4 Electrophysiology

For electrophysiological recordings, cells grown on coverslips were used 24h after transfection. The coverslips with attached cells were transferred into an experimental chamber mounted on the stage of an inverted Axiovert 200 microscope (Zeiss, Oberkochen, Germany). Experimental chamber was filled with a bath solution of the following composition (in mM): 140 NaCl, 10 HEPES, 10 Glucose, 2 MgCl_2_, 2 CaCl_2_, pH adjusted to 7.4 with NaOH. Positively transfected cells were identified by green or red fluorescence when illuminated using CoolLED pE-300 ultra (Scientifica, Great Britain). The *cis*- isomerization of OptoBI-1 was induced by illumination at 365 nm during 1, 5 or 15 sec (depending on the protocol).

Transmembrane currents were recorded using the whole-cell configuration of the patch-clamp technique in the voltage-clamp mode, as we described previously (39, 40). Patch-pipettes were fabricated from thin-wall filament glass capillaries (Harvard Apparatus, W3 30-0068) using a vertical puller (Sutter Instruments P1000 puller, USA). After being filled with the solution containing (in mM): 120 cesium methanesulfonate, 20 CsCl, 15 HEPES, 5 MgCl_2_, 3 EGTA, titrated to pH 7.3 with CsOH, the patch pipettes had a resistance 2–4 MΩ.

Once the whole-cell configuration was obtained, the stock solution of OptoBI-1 was added to the bath solution to reach the final concentration of 10 µM. Recordings were started about 1.5 minutes afterwards under no-flow conditions.

Whole-cell current recordings were conducted using an Axopatch 200B amplifier (Molecular Devices, USA) coupled to a Digidata-1550B Digitizer (Molecular Devises, USA). Whole-cell currents were normalized to current density (pA/pF) by dividing by cell capacitance. Experiments were performed at room temperature.

Data collection and analysis were performed using Clampex and Clampfit software, respectively, of the pCLAMP software suite (version 11.2, Axon Instruments, USA).

### 4.5 Confocal microscopy

RBL-2H3 cells were kept in Tyrode’s buffer (as described above) before and during confocal imaging.

High-resolution imaging was performed with an array confocal laser scanning microscope (Axiovert 200 M, Zeiss) with a 100×/1.45 NA oil immersion objective (Plan-Fluor, Zeiss) and a Nipkow-based confocal scanner unit (CSU-X1, Yokogawa Electric Corporation, Tokyo, Japan). Laser light of diode lasers (Visitron Systems) served as the excitation light source. BFP-KDEL was excited at 405 nm, TRPC6-CFP at 445 nm, pH-Lemon-GPI at 514 nm, and the RFP fusion constructs (TRPC6-RGECO, mApple-Farnesyl-5, mCh-ER3) at 561 nm. Emissions were captured with a CoolSNAP HQ2 CCD Camera (Photometrics, Tucson, Arizona, USA) using the following emission filters (Chroma Technology Corporation, VT, USA): ET460/50 m for BFP-KDEL, ET480/40 m for TRPC6-CFP, ET525/36 m for pH-Lemon-GPI, and ET630/75 m for the RFP-based fusion constructs. Image acquisition was done with VisiView Premier Acquisition software (Visitron Systems). Image analysis was performed with ImageJ software.

### 4.6 Statistical analysis and data correction

Statistical data are shown as mean ± SEM. Comparison of multiple groups was performed using analysis of variance (ANOVA) and Dunnett’s *post hoc* test. The significance levels are as follows: ns P > 0.05, * P ≤ 0.05, ** P ≤ 0.01, *** P ≤ 0.001, **** P ≤ 0.0001.

Due to a photobleaching effect manifesting in the form of a signal drop below the baseline (Ca^2+^ imaging experiments), a semi-automated baseline correction process was performed (when required) using the Origin Pro 2018 software (OriginLab Corporation, Northampton, MA, USA).

## Data Availability

The raw data supporting the conclusions of this article will be made available by the authors, without undue reservation.
