# Supplementary material for: Photopharmacology reveals high-specificity linkage of Ca2+ entry at TRPC6 nanodomains to NFAT activation in mast cells
Source: Front Immunol. 2025 Jul 24;16:1595036. doi: 10.3389/fimmu.2025.1595036 (PMC12329589; doi:10.3389/fimmu.2025.1595036)
Supplement: Supplementary file 1 [file DataSheet1.pdf]

Supplemental Material

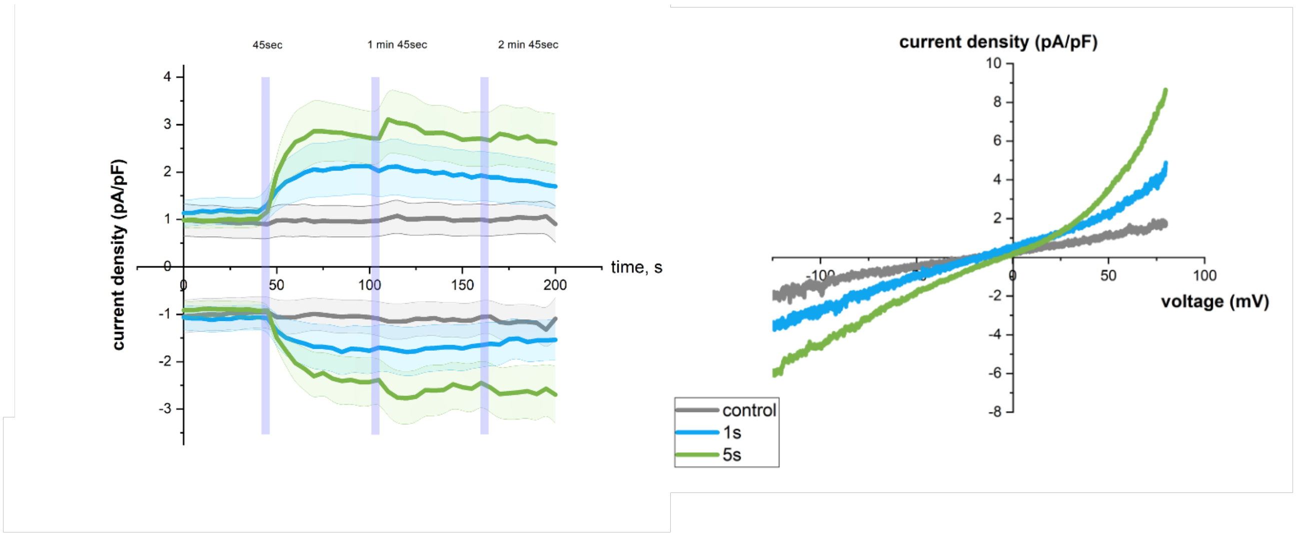

Suppl. Fig. 1: Photopharmacological activation of TRPC6 currents in RBL-2H3 cells. A Time courses of conductances recorded at −90 to +70 mV during repetitive photoconversion of OptoBI-1, which are induced by light at 365 nm. The effect of the light lasts either 5s for control (grey, n = 13 cells) and TRPC6-YFP cells (green, n = 13 cells) or 1s for TRPC6-YFP cells (blue, n = 15 cells). **B** Representative I/V relations of OptoBI-1-induced currents in control and YFP-TRPC6 transfected RBL-2H3 cells applying voltage-ramp protocols. The effect of the light (365nm) lasts either 5s for control (grey) and TRPC6-YFP cells (green) or 1s for TRPC6-YFP cells (blue).
