# Supplementary material for: Photopharmacology reveals high-specificity linkage of Ca2+ entry at TRPC6 nanodomains to NFAT activation in mast cells
Source: Front Immunol. 2025 Jul 24;16:1595036. doi: 10.3389/fimmu.2025.1595036 (PMC12329589; doi:10.3389/fimmu.2025.1595036)
Supplement: Supplementary file 2 [file DataSheet2.pdf]

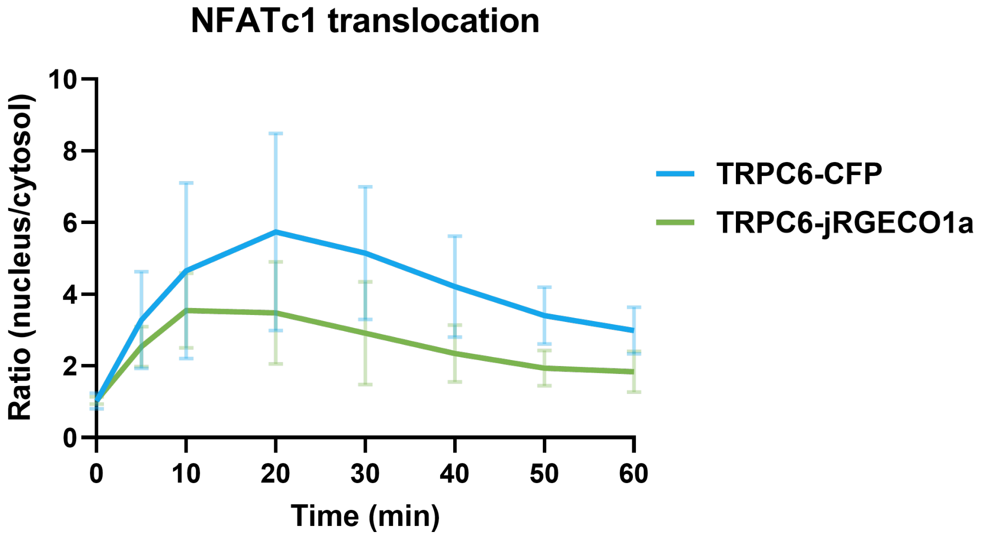

Suppl. Figure 2: Light-controlled TRPC6-activation reaches the maximum of NFATc1 nuclear translocation within 10-20 min in RBL-2H3 cells.

Statistical representation of NFATc1 nuclear translocation triggered by 3x15 s illuminations with UV light (365 nm, not depicted) 25s after addition of the photochromic TRPC3/6/7 activator OptoBI-1 (30 µM) over the course of 60 min. NFATc1 activation is quantified as the nucleus:cytosol fluorescence ratio of the co-expressed NFATc1 reporter fusion protein. RBL-2H3 cells were genetically modified to overexpress TRPC6-YFP (green; n=5) along with NFATc1-mCherry as a reporter for transcriptional activation. TRPC6-jRGECO1a (blue; n=8) was expressed together with NFATc1-YFP. All experiments were performed in the presence of 2 mM extracellular Ca^{2+}.
